# Suppressing Antibacterial Resistance: Chemical Binding of Monolayer Quaternary Ammonium Salts to Polymethyl Methacrylate in an Aqueous Solution and Its Clinical Efficacy

**DOI:** 10.3390/ijms20194668

**Published:** 2019-09-20

**Authors:** Chung-Yuan Lee, Yi-Ting Chen, Bor-Shiunn Lee, Che-Chen Chang

**Affiliations:** 1 Department of Chemistry, National Taiwan University, No. 1, Sec. 4, Roosevelt Road, Taipei 10617, Taiwan; r01223218@ntu.edu.tw; 2 Graduate Institute of Oral Biology, School of Dentistry, National Taiwan University and National Taiwan University Hospital, Taipei 10048, Taiwan; r02450005@gmail.com

**Keywords:** polymethyl methacrylate, quaternary ammonium salts, covalent binding, monolayer surface bonding, Fourier transform infrared difference spectroscopy, concordant alignment, antibacterial activity, biocompatibility, antibacterial resistance

## Abstract

Antibacterial resistance (ABR) poses an enormous threat to human health. ABR mainly develops due to bacteria being constantly exposed to diluted levels of disinfectants. Here, we propose a method for suppressing ABR through the chemical binding of disinfectants to polymethyl methacrylate (PMMA) device surfaces in solutions of 5%, 10%, and 20% disinfectant concentrations. PMMA discs were fabricated from a commercial orthodontic acrylic resin system (Ortho-Jet) and quaternary ammonium salts (QAS), 3-(trimethoxysilyl)-propyldimethyloctadecyl ammonium chloride (42% in methanol), were used as the disinfectant. The PMMA surfaces were activated in 3 M sulfuric acid at 80 °C for 5 h for the esterification of hydrolyzed QAS to PMMA. Fourier transform infrared difference spectra confirmed that the carboxy-terminated PMMA was chemically bound to the QAS. In vitro cell viability tests using 3-(4,5-dimethylthiazol-2-yl)-2,5-diphenyl tetrazolium bromide assays revealed that 5%QAS-c-PMMA was more biocompatible than 10%QAS-c-PMMA and 20%QAS-c-PMMA. The results of antibacterial tests and clinical trials demonstrated the excellent antibacterial power of 5%QAS-c-PMMA. This method is the first solution-based approach to successfully avoid disinfectant leakage and subsequent ABR, as revealed by mass spectrometry studies of the solution obtained by agitating the disinfectant-bound PMMA for 28 days.

## 1. Introduction

The evolution of antibacterial resistance (ABR) in bacterial pathogens [1,2] and the emergence of antibiotic-resistant bacterial strains [3] have caused increasingly severe threats to global public health [4,5]. Resistance has been observed against nearly all antibiotics [1]. Initial research conducted by a review team commissioned by the British prime minister revealed that antimicrobial resistance will become the leading cause of death by 2050, with 10 million deaths per year at a cost to the world GDP of 100 trillion USD [6]. Other than the overuse of antibiotics in medication and animal husbandry, the antibiotic resistance crisis may be partially attributable to the increasing use of biomaterials and indwelling medical devices [7,8]. Opportunistic pathogens such as coagulase-negative *Staphylococci* and *Pseudomonas aeruginosa* can cause infections in patients with implanted medical or prosthetic devices [7,9,10].

Polymethyl methacrylate (PMMA) and its composites are the predominant biomaterials used for indwelling devices, which are loaded with antibiotics or disinfectants as a standard procedure for antibacterial treatment [11,12]. Like other biomaterials, PMMA carries the risk of microbial colonization and infection. Two separate hospital studies conducted in Canada and Japan [13,14] over a period of approximately 20 years revealed that complications resulting from infection in patients using PMMA are more common than the average infection rate for patients treated with various biomaterials. Most antibacterial PMMA biomaterials loaded with antibiotics are processed using bulk mixing methods involving disinfectants [15,16,17,18]. To reduce the release of loaded disinfectants from the processed PMMA and the subsequent development of antimicrobial resistance, different approaches have been reported for grafting antibiotics to PMMA surfaces. They include (1) the swelling penetration of Ag or Au nanoparticles by pouring a tetrahydrofuran solution onto PMMA films [19]; (2) atomic layer deposition of ZnO films on PMMA substrates using diethylzinc and water precursors [20]; (3) stirring grafting of Ag nanoparticles encapsulated with polymers with surface functional groups to PMMA supports [2]; (4) deposition of chitosan-stabilized disinfectant nanoparticles on PMMA using a heterocoagulation technique [21]; (5) repeated dip-coating of PMMA slides with dendritic porous nanostructures embedded with disinfectant nanoparticles before organic vapor treatment [22]; and (6) plasma activation of PMMA surfaces with subsequent immersion of the activated samples into a disinfectant solution [23,24]. These strategies allow disinfectants to be grafted onto the PMMA surface and the resulting product is abbreviated as disinfectant-g-PMMA herein. For disinfectant-g-PMMA, the antibacterial agents loaded on the PMMA surface are not all covalently bonded to PMMA. The non-bonded agents are prone to release from the fabricated biomaterials [25]. The minute release of antibacterial agents can contribute to the development of bacterial resistance to them [26,27], even though different groups of bacteria vary in their susceptibility to the agents [28]. The release of antibacterial agents with a low reactivity also leads to their accumulation in the environment, such that bacterial resistance develops through the prolonged exposure of bacterial populations to diluted levels of the agents [26,27,29]. Of particular concern is the release of agents that can cause bacteria to acquire resistance through alterations of their genetic composition [25]. Studies of bacterial resistance to antibacterial agents such as quaternary ammonium salts (QAS) have shown a loss of resistance upon removal of the agents from the growth media [30,31]. Resistance to QAS genetically develops through mutation and plasmid encoding [32]. Without careful control, the transfer of encoded plasmids arising from the omnipresence of diverse antibacterial agents resulting from overuse may promote the spread of multi-drug-resistant plasmids [33].

Instead of surface grafting, we propose a method for suppressing ABR through the chemical binding of disinfectants to PMMA device surfaces with covalent bonds in aqueous solutions of appropriate disinfectant concentrations. The resulting covalently bonded product is abbreviated as disinfectant-c-PMMA herein. The covalent binding of the disinfectant to the biomaterial surface inhibits disinfectant release, even under aberrant conditions, and curtails the problem of a disinfectant overdose in bulk manufacturing. The suppression is achieved because any bacteria in the solution are in a completely disinfectant-free region if they are far from the PMMA and if they approach it, they are killed by the high-concentration, parallel-aligned disinfectant molecules bonded to the PMMA surface. Disinfectant surface binding also allays concerns about the possible structural fragility of materials and the selection of resistant mutants under a prolonged release of loaded disinfectants [34]. Among disinfectants, quaternary ammonium salts (QAS) are potent candidates for imbuing PMMA biomaterials with superior antibacterial properties. They are bactericidal, fungicidal, and virucidal against enveloped viruses (e.g., HIV) and have an impressive regenerability and low cytotoxicity to human cells [35]. The covalent binding of QAS to the PMMA surface was characterized in this study via Fourier transform infrared (FTIR) difference spectroscopy. The antibacterial power of the QAS-bound PMMA was assessed using optical density measurement. For an examination of the cell viability of the PMMA surface-bound QAS, a 3-(4,5-dimethylthiazol-2-yl)-2,5-diphenyl tetrazolium bromide test was performed. Clinical trials were conducted using confocal laser scanning microscopy to evaluate the colonization of microorganisms on the sample. The release of QAS from the QAS-bound PMMA in the solution was investigated by bathing and agitating the sample in deionized water for an extended period of time and through detection using mass spectrometry.

## 2. Results

### 2.1. QAS Binding to PMMA

**Figure 1:**
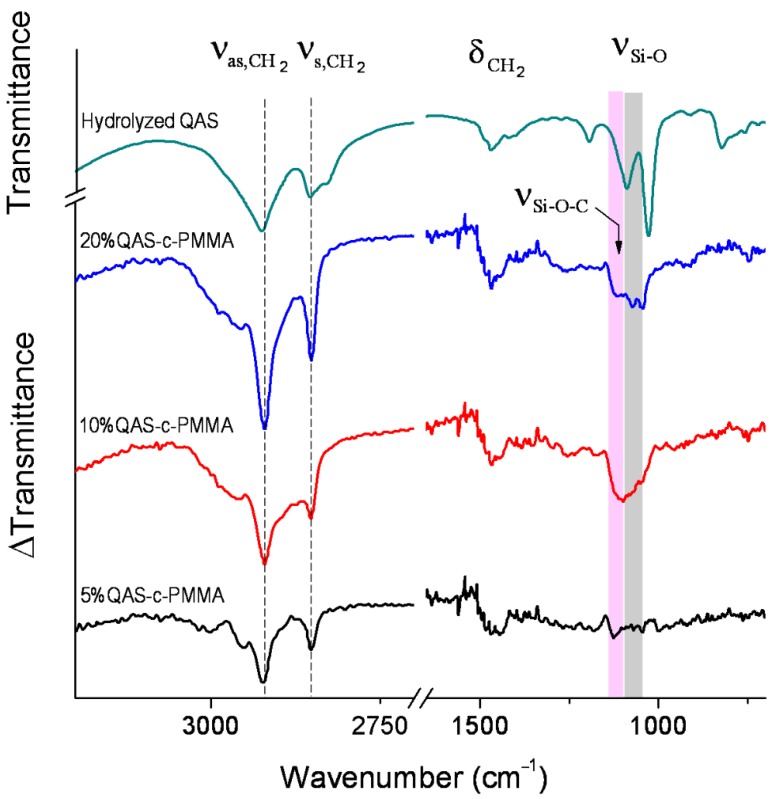
Fourier transform infrared (FTIR) difference spectra (relative to the acid-treated polymethyl methacrylate (PMMA), using the absorbance of the peak at 1721 cm^−1^ as a reference), examined using attenuated total reflectance, of the QAS-c-PMMA samples prepared in 5%, 10%, and 20% quaternary ammonium salts (QAS) solutions, and the FTIR spectrum of the hydrolyzed QAS, measured in transmission mode. All spectra were obtained at a 4 cm^−1^ resolution. The bands at 2826 and 1027 cm^−1^ in the hydrolyzed QAS spectrum were mainly associated with the methanol in the QAS solution [40,41]. The pink shading highlights the wavenumber region for the absorption band of Si-O-C, as also indicated by an arrow, whereas the grey shading highlights that of Si-O-Si and the unbound silanol groups of QAS. Dashed vertical lines are to guide the eyes for the absorption bands at different QAS concentrations due to CH_2_ stretching and in-plane deformation vibrations.

High-performance liquid chromatography/mass spectrometry (HPLC/MS, LCMS-8060, Shimadzu, Kyoto, Japan) revealed no QAS in the solution obtained by immersing and agitating QAS-c-PMMA in distilled water for 28 days. Fourier transform infrared difference spectra (Figure 1), obtained by subtracting the unbound, acid-treated PMMA spectrum from the individual QAS-c-PMMA spectra, revealed that QAS were chemically bonded to PMMA, forming an Si-O-C bond. The stretching of this bond contributed to the absorption bands observed at 1130−1090 cm^−1^ (pink shading) in the difference spectra [36], as evidenced by the sensitivity to the hydrolysis of these bands in the spectra taken from Si-O-C structures, such as tetraethyl orthosilicate. The bands at 1085−1030 cm^−1^ (grey shading) characterized the unbound silanol groups of QAS [37] and were also ascribed to antisymmetric stretching of the Si-O-Si bonds [38] formed by the irreversible cross-linking of QAS molecules, because silanol groups are highly reactive [39]. The Si-O-C bands confirmed that QAS were covalently bonded to the carboxy site of PMMA. Because of the higher alkyl proportion in QAS compared with PMMA, the bonding also caused intensity increases with corresponding increases in QAS concentrations in the QAS-c-PMMA absorbance difference spectra for CH_2_ stretching and in-plane deformation vibrations at 2935−2840 cm^−1^ and 1500−1425 cm^−1^, respectively [40].

### 2.2. Antibacterial Tests

**Figure 2:**
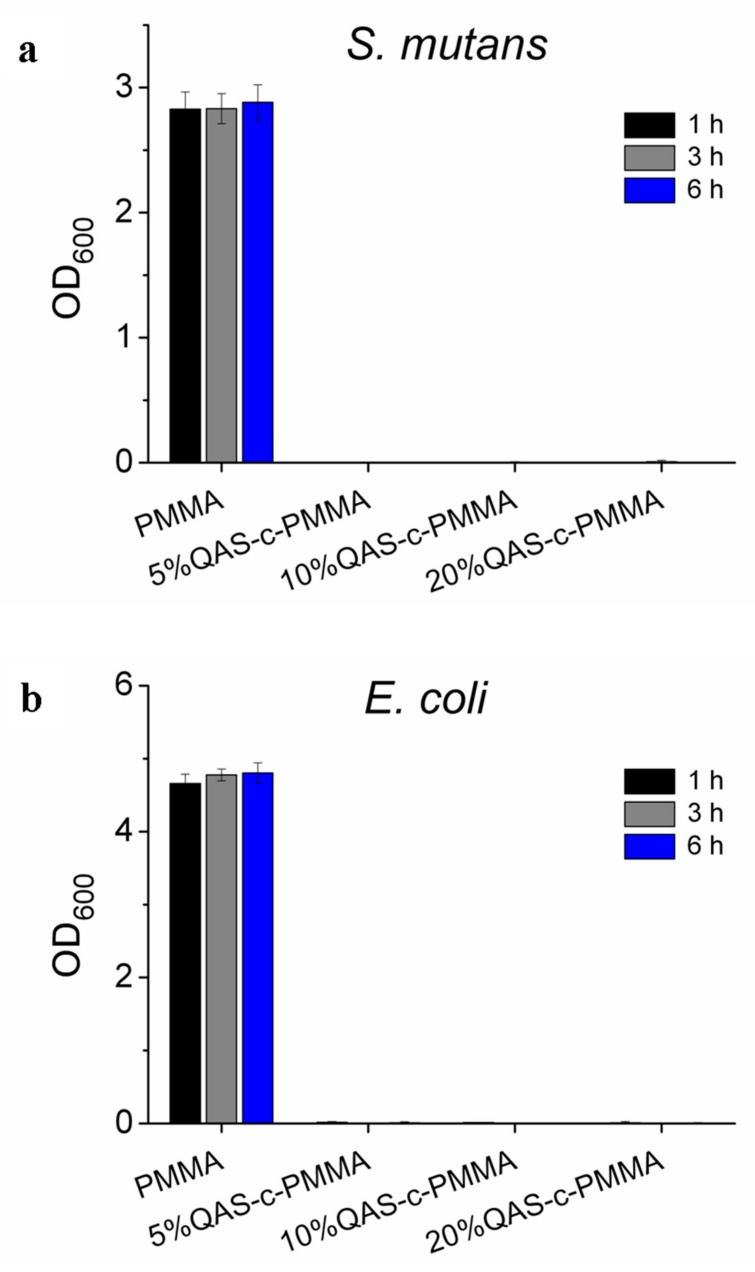
Antibacterial adhesion tests: (**a**) *Streptococcus mutans* and (**b**) *Escherichia coli*. All PMMA chemically bonded to QAS demonstrated antibacterial adhesion.

Antibacterial adhesion tests indicated that the PMMA chemically bonded with QAS exhibited excellent antibacterial activity against both *Streptococcus mutans* and *Escherichia coli*. The optical density of the culture medium, measured at 600 nm (OD_600_), showed that bacteria were more heavily suppressed on QAS-c-PMMA compared with PMMA (Figure 2) for QAS concentrations as low as 5%. The suppression occurred independent of the bacteria adhesion period tested.

### 2.3. Viability Tests

**Figure 3:**
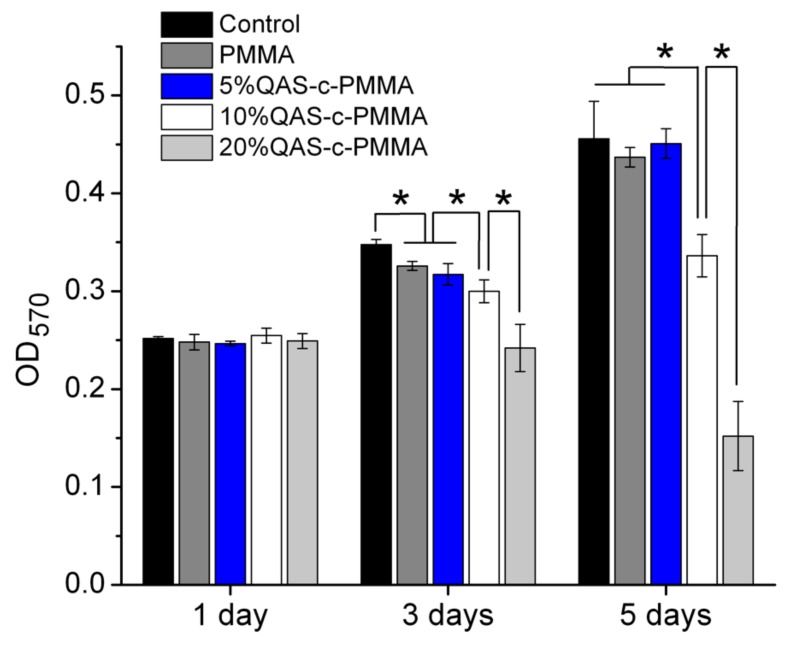
Cell viability test of PMMA, 5%QAS-c-PMMA, 10%QAS-c-PMMA, and 20%QAS-c-PMMA. The QAS-c-PMMA plates were placed at the bottom of Transwell inserts (diameter 6.5 mm, pore size 3.0 μm) before the inserts were transferred to and incubated in 24-well culture plates, in which human gingival fibroblasts (HGF) at 5 × 10^4^ cells per well had been seeded overnight at 37 °C. Empty Transwell inserts served as a negative control group. After incubation for 1, 3, and 5 days, a 3-(4,5-dimethylthiazol-2-yl)-2,5-diphenyl tetrazolium bromide test was performed to examine the cell viability. * *p* < 0.05.

In vitro cell viability tests (NTUH201105080RC) using primary cultures of human gingival fibroblasts (HGF) showed that 5%QAS-c-PMMA was biocompatible with the HGF. The optical density (OD_570_) of the test solution, measured using a microplate reader (ELx 800, BioTek, VT, USA), showed no major difference in cell viability among all specimen groups after 1 day of culture (Figure 3). After 3 and 5 days of culture, 5%QAS-c-PMMA demonstrated a far lower cytotoxicity than 10%QAS-c-PMMA and 20%QAS-c-PMMA. Moreover, no major difference was observed in cytotoxicity between 5%QAS-c-PMMA and the control groups after 5 days of culture.

As discussed, at 10% QAS and higher, the fabricated QAS-c-PMMA had poorly ordered QAS layers, which were less stable. The Si-O-Si bonds between the randomly packed QAS might be hydrolyzed [42] during prolonged viability tests, releasing their long alkyl chains from entanglement. The free hydrophobic chains could permeate into the hydrophobic cell membrane [43], resulting in some cytotoxicity for fibroblasts (Figure 3) and keratinocytes [44].

### 2.4. Clinical Trials

**Figure 4:**
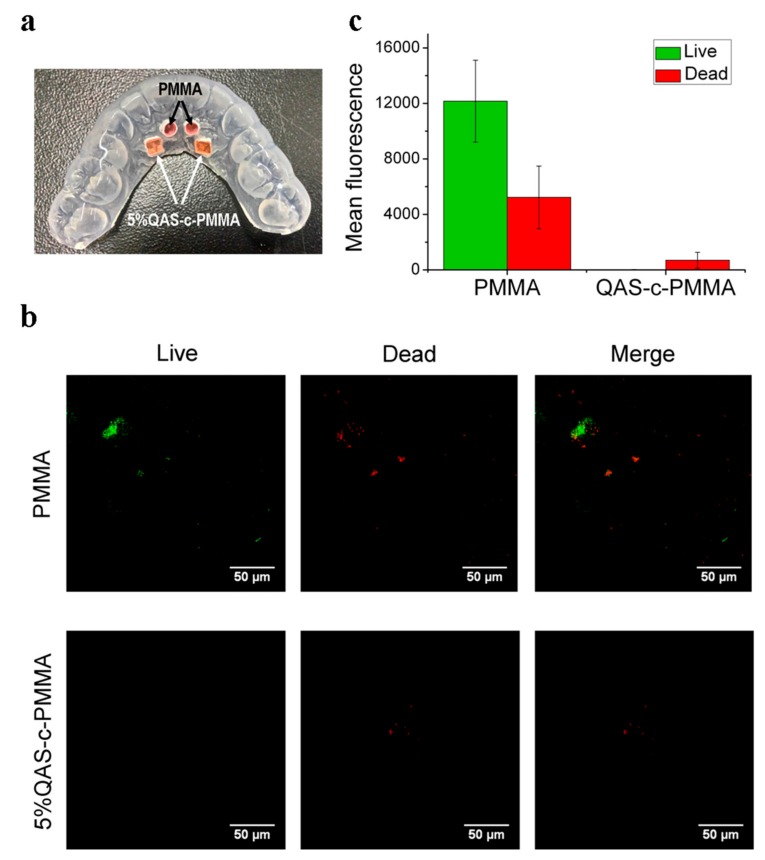
Confocal laser scanning microscope (CLSM) live/dead fluorescent staining tests: (**a**) Removable acrylic appliance holding two PMMA and two 5%QAS-c-PMMA discs; (**b**) CLSM images obtained from PMMA (top row) and 5%QAS-c-PMMA (bottom row) discs with dead bacteria fluorescing red from propidium iodide (PI) staining and live bacteria fluorescing green from SYTO 9 staining; (**c**) the total cell fluorescence of the stained cells averaged over the CLSM images, after correction against the mean fluorescence of background readings in the area of the selected cells, obtained from all PMMA and 5%QAS-c-PMMA discs.

Bacteria adhered to surfaces, growing as biofilms, are known to be considerably less susceptible to antibiotics than planktonic forms of the same bacteria [45]. Antibacterial agents fixed within resins, such as methacrylate, have been reported as having a minimal effect on biofilm formation [46]. A clinical trial, approved by the Research Ethics Committee of National Taiwan University Hospital (NTU201411031DIND, NCT02634996), was thus performed to evaluate the antibacterial effect of the chemically bonded QAS on biofilm formation on the bonded PMMA surface. Written informed consent was obtained from all of the participants prior to the study. Confocal laser scanning microscope (CLSM, TCS SP5, Leica, Wetzlar, Germany) images (Figure 4b) showed that far fewer bacteria were detected on the 5%QAS-c-PMMA than PMMA.

The fluorescence intensities of the stained dead and live cells were identified using Image J (Java, National Institutes of Health, MD, USA) based on a random selection of three CLSM images obtained from each sample surface [47]. Overall, the 5%QAS-c-PMMA surfaces displayed far less fluorescence than PMMA, confirming that the chemically bonded QAS effectively prevented microorganisms from colonizing on the 5%QAS-c-PMMA surface (Figure 4c). Moreover, ultimate bactericidal activity was achieved on the 5%QAS-c-PMMA surface, on which few bacteria survived. The results revealed that QAS may function effectively, even when chemically bonded to biomaterial surfaces. The large intensity difference shown in Figure 4c, despite the PMMA and QAS-c-PMMA specimens on the mouthguard being exposed to environments of an identical bacterial concentration, suggests that the chemically bonded QAS exhibit scavenging activity on dead bacterial cells. Of the bacteria killed by the chemically bonded QAS, which ought to be far outnumbered on QAS-c-PMMA compared with PMMA, very few remained on the QAS-c-PMMA.

## 3. Discussion

The absence of the 1130−1030 cm^−1^ feature in the difference spectrum of 5%QAS-c-PMMA (Figure 1) also indicated the covalent binding of QAS to PMMA. At a low QAS concentration, the bulky QAS molecules were relatively free to approach PMMA and esterification occurred between the silanol of the hydrolyzed QAS and the carboxy of the acid-activated PMMA. The resulting Si-O-C covalent bonds were inclined toward the PMMA surface, allowing long alkyl chains of QAS to be concordantly aligned in parallel in a more upright fashion for stabilization [48]. Given the inclined bonding configuration, the stretching vibrations of the Si-O-C bonds were IR inactive because dipole moment derivative components perpendicular to the surface were almost nonexistent [49]. A monolayer of QAS concordantly aligned and bound to PMMA has a thickness of approximately 2.5 nm [50]. At 10% QAS and higher, the concentration was above the threshold for the free approach to form monolayer QAS on PMMA. Instead, when approaching PMMA, the bulky QAS molecules in higher-concentration solutions restrained one another from binding to PMMA in the necessary orientation, such that the formed Si-O-C bonds were not all fully inclined to the surface [39,51]. This produced the non-zero absorbance of the 1130–1030 cm^−1^ feature shown in Figure 1. The resulting alkyl chains were packed in tangled and poorly ordered layers [51]. The surface attachment of QAS thus reached saturation at a QAS concentration below 10%.

The greater suppression of bacterial growth on QAS-c-PMMA compared with that of the growth on PMMA (Figure 2) may have been partially caused by the polycations present on QAS-c-PMMA. As discussed, the long alkyl chains of the QAS in 5%QAS-c-PMMA were concordantly aligned in parallel. They were stabilized by the network of Si-O-Si bonds between chains (Figure 1). The positively charged nitrogen of QAS remained at the surface of QAS-c-PMMA and acted like polycations, which may have disrupted bacterial metabolism by creating a charge imbalance on the cell membrane of the bacteria on contact. For example, charged or polar residues in the h-region of the cleavage site of an N-terminal signal sequence, which leads to protein export in both prokaryotic and eukaryotic cells, often reduce the export [52]. Because prokaryotic proteins have a considerably higher incidence of acidic residues than basic residues around the cleavage site, polycations can inhibit the formation of a net positive charge at the site in bacteria cells [53], resulting in export-defective signal sequence mutations [53,54].

The scavenging activity of the chemically bonded QAS on dead bacterial cells (Figure 4c) conferred on the QAS-c-PMMA system the potential of long-term antibacterial activity. For example, PMMA is widely used in dentistry as a material for temporary crowns, removable partial dentures, and orthodontic retainers. QAS-c-PMMA could suppress the induction of caries or periodontitis in the surrounding teeth.

## 4. Materials and Methods

### 4.1. Preparation of PMMA Discs

An auto-polymerizing methyl methacrylate/poly (methyl methacrylate) (MMA/PMMA) orthodontic acrylic resin system (Ortho-Jet; Lang Dental Manufacturing Co. Inc., IL, USA) was used to fabricate PMMA discs. In the fabrication process, 0.1 g of PMMA powder was mixed with 0.1 mL of MMA monomers, and 5 mm diameter and 1 mm thick discs were produced by placing the mixture in stainless steel molds. After gelling, PMMA discs were placed in Aquapres (Lang Dental Manufacturing Co. Inc.) at 1.5 kg/cm^2^ for 1 h.

### 4.2. Binding of QAS to PMMA

Among QAS, 3-(trimethoxysilyl)-propyldimethyloctadecyl ammonium chloride (42% in methanol, Sigma-Aldrich, MO, USA) was used in this study. To chemically bond QAS to PMMA, the surface of PMMA plates was activated using treatment in 3 M sulfuric acid (Fisher Scientific, PA, USA; 98%) at 80 °C for 5 h to produce carboxy-terminated PMMA. The QAS were hydrolyzed by replacing methanol in a QAS stock solution with deionized water (18.3 MΩ-cm) to convert their silicic acid ester to silanol. Subsequent heating of the carboxy-terminated PMMA at 80 °C in the presence of the hydrolyzed QAS enabled esterification to occur, chemically bonding the QAS to PMMA, and thus forming QAS-c-PMMA.

### 4.3. Analysis of QAS Released from QAS-c-PMMA into the Solution

Three QAS-c-PMMA discs were immersed in distilled water and agitated for 28 days. HPLC/MS analyses of the chemicals released into the solution were performed using an LC-MS/MS 8060 system (Shimadzu, Kyoto, Japan), which consists of a Shimadzu Nexera ultra high-performance liquid chromatography (UPLC) system coupled with a triple quadrupole mass spectrometer. The UPLC system was equipped with two LC-30 AD pumps, a CTO-30Ac column oven, and an SIL-30AC auto-sampler. LabSolutions LCMS Ver.5.80 software (Shimadzu, Kyoto, Japan) was used for system control, data collection, and quantitation.

Chromatographic separation was achieved on a Shim-pack XR-ODS column (2.0 mm I.D. × 75 mm, 2.2 μm). For QAS analysis, the optimized gradient elution consisted of 0.01% formic acid in water (mobile phase A) and 0.01% formic acid in methanol (mobile phase B), both in a 15 mM ammonium acetate buffer. The flow rate was 0.35 mL/min. The elution gradient program started with an initial mobile phase composition of 65% B. The composition was increased in a linear fashion to 95% B over 1 min, then held constant for 3 min, and finally brought back to the initial condition of 65% B over 0.10 min, followed by a 3-min re-equilibration. During the elution, the column oven was maintained at 35 °C. The injection volume of all samples was 5 μL.

The triple quadrupole mass spectrometer in the LC-MS/MS 8060 system was capable of electro-spray ionization and multiple reaction monitoring (MRM) detection. The MS parameters were optimized to obtain the most sensitive and specific MS transition for QAS by the direct infusion of 1 mM of the standard solutions into the ion source with a syringe pump. The optimized parameters for MRM detection in the positive mode were as follows: nebulizer gas: 3.0 L/min; heating gas: 10 L/min; drying gas: 10 L/min; interface temperature: 300 °C; desolvation line temperature: 250 °C; and heat block temperature: 400 °C. The dwell time was set to 100 ms. Table 1 shows the MRM transition selected for monitoring QAS and the compound-dependent parameters for QAS, such as voltage potential Q1 and Q3, and the collision energy (CE).

### 4.4. FTIR Difference Spectroscopy

All FTIR spectra were measured using a Jasco 4200 spectrometer equipped with a deuterated lanthanum -alanine-doped triglycine sulfate (with Peltier temperature regulation) detector. Prior to each measurement, the attenuated total reflection crystal was cleaned with 95% ethanol (Sigma-Aldrich, MO, USA). The crystal was then cleaned with methanol (Fisher Scientific; 99.99% analytical grade, Waltham, MA, USA) and purged with nitrogen until there was no noticeable change in the background spectra. Each spectrum was typically constructed from 100 scans (approximately 1.5 min of total acquisition time), with a spectral resolution of 4 cm^−1^. The spectrum was subtracted from the background spectrum recorded immediately before sample illumination. The difference spectrum was then calculated relative to the acid-treated PMMA spectrum, using the absorbance of the peak at 1721 cm^−1^ as a reference.

### 4.5. Antibacterial Test

Gram-positive *S. mutans* and Gram-negative *E. coli* stored at −80 °C were cultured on tryptic soy agar (TSA; BD Biosciences, NJ, USA) at 37 °C overnight separately. A strain of a single colony on TSA was then cultured in 10 mL of tryptic soy broth (TSB; BD Biosciences) at 37 °C with an orbital shaker incubator at 220 rpm for 16 h. After 16 h of culture, the *S. mutans* and *E. coli* strains were adjusted to a concentration of 10^7^ colony forming units/mL (OD_600_ = 0.2) before use. The bacterial suspension (OD_600_ = 0.2, 50 μL) was dropped on sterilized QAS-c-PMMA plates after the plates were incubated in pooled sterile saliva [55] for 1 h at 37 °C. The bacteria were then incubated on QAS-c-PMMA plates at 37 °C for 1, 3, and 6 h (*n* = 6) for adhesion. After 1, 3, and 6 h, the plates were washed with sterile phosphate buffered saline (Lonza Walkersville, Inc. Walkersville, MD, USA) and cultivated at 37 °C for 24 h. The culture medium was measured at the OD_600_ value.

### 4.6. Cell Viability Assay

Primary cultures of human gingival fibroblasts (HGFs) were used. The trial was approved by the Research Ethics Committee of National Taiwan University Hospital (8 June 2011) and was registered as Case No. 201105080RC. Written informed consent was obtained from all participants before the collection of gingival tissues. HGFs were cultured in minimum essential medium alpha with 10% fetal bovine serum and 100 U/mL of antibiotics (penicillin–streptomycin–amphotericin; Sigma-Aldrich, MO, USA) at 37 °C in 5% CO_2_. The passage number was 8–12. The specimens in each group (*n* = 5) were placed at the bottom of Transwell inserts (Costar Transwell Permeable Supports, NY, USA; diameter 6.5 mm, pore size 3.0 mm). For a comparison of the relative toxicities of the tested materials, the Transwells were transferred into 24-well culture plates, which had been seeded with HGFs at 5 × 10^4^ cells per well and allowed to adhere overnight at 37 °C. Empty inserts served as a negative control group. After incubation for 1, 3, and 5 days, the cells in each well were incubated at 37 °C for 3 h with culture medium containing 100 µL of 3-(4,5-dimethylthiazol-2-yl)-2,5-diphenyl tetrazolium bromide solution (Sigma-Aldrich, MO, USA; 98%). The medium was then aspirated and 200 µL of dimethyl sulfoxide (Sigma-Aldrich, MO, USA; ≥99.9%) was added to dissolve the reduced formazan crystals. The optical density (OD_570_) of the formazan solution was measured using a microplate reader (ELx 800, Biotek, VT, USA).

### 4.7. Clinical Trial

A clinical trial was performed to evaluate the antibacterial effect of the chemically bonded QAS on biofilm formation on the bonded PMMA surface. After mouthguards were sterilized using ultraviolet irradiation for 12 h, two PMMA discs, used as a control group, and two 5%QAS-c-PMMA discs, were mounted on a sterilized mouthguard for each participant (*n* = 10) to wear in the trial (Figure 4a). Each participant wore the mouthguard on the upper jaw and maintained regular activity during the experiment. After 5 h of wear, the discs were removed from the mouthguard and washed using PBS three times before staining for 15 min (protected from light) using a LIVE/DEAD BacLight bacterial viability kit (Invitrogen, OR, USA). The kit, which consists of PI and SYTO^®^ 9, was used to analyze the adherence and viability of bacteria on the samples using a CLSM (TCS SP5, Leica, Wetzlar, Germany). The excitation and emission wavelengths used were 480 and 500 nm for the live bacteria and 490 and 635 nm for the dead bacteria, respectively.

## 5. Conclusions

A method for suppressing ABR through the confinement of monolayer disinfectants on biomaterial surfaces via chemical bonding has been proposed in this paper. Accordingly, a simple synthetic route that chemically binds monolayer QAS to PMMA surfaces in an aqueous solution to form QAS-c-PMMA has been demonstrated. The antibacterial efficacy/cell viability findings and the null detection by HPLC/MS of the QAS in the solution obtained by bathing and agitating QAS-c-PMMA in deionized water for 28 days prove the efficacy of our proposed method. The efficacy was achieved partially because of the parallel alignment of QAS chemically bonded to PMMA surfaces. The parallel alignment was achieved through self-assembly in solutions of appropriate QAS concentrations. The results also substantiated the chemical interaction of QAS with the PMMA surface in the solution. Despite QAS having been one of the most effective disinfectants for more than a century and PMMA having the longest scientific history as a biomaterial, this is the first report of this approach of chemically bonding them, which provides a powerful method for enabling the coupling of QAS with PMMA in a solution to achieve ultimate bactericidal activity for PMMA medical devices. The resulting QAS coating on the PMMA surface only has a single-digit nanometer thickness. Most importantly, the proposed method is the first solution-based approach to completely avoid disinfectant leakage from the disinfectant-loaded biomaterial surface and the subsequent development of ABR. The method is thus environmentally friendly and may be further developed for home use.

## Figures and Tables

**Table 1:** Mass spectrometric parameters for QAS: precursor to fragment ion transition, voltage potential (Q1), collision energy (CE), and voltage potential (Q3).

Analytes	MRM Transition *m*/*z* (Q1→Q3)	Q1 (V)	CE (V)	Q3 (V)	Retention Time
**QAS**	460.3→121.05	−20	−36	−20	4
